# The smallest biggest theropod dinosaur: a tiny pedal ungual of a juvenile *Spinosaurus* from the Cretaceous of Morocco

**DOI:** 10.7717/peerj.4785

**Published:** 2018-05-30

**Authors:** Simone Maganuco, Cristiano Dal Sasso

**Affiliations:** 1 Museo di Storia Naturale di Milano, Milano, Italy

**Keywords:** *Spinosaurus*, Cenomanian, Theropoda, Pedal ungual, Morocco

## Abstract

We describe a nearly complete pedal ungual phalanx, discovered in the Kem Kem Beds (Cenomanian) of Tafilalt region, south-eastern Morocco. The bone is symmetric, pointed, low, elongate, and almost flat ventrally in lateral aspect. This peculiar morphology allows to refer the specimen to the smallest known individual of the genus *Spinosaurus*. The bone belongs to an early juvenile individual and it is proportionally identical to the ungual of the third digit of a large partial skeleton recently found, suggesting an isometric growth for this part of the pes and the retention of peculiar locomotor adaptations—such as traversing soft substrates or paddling—during the entire lifespan.

## Introduction

**Figure 1:**
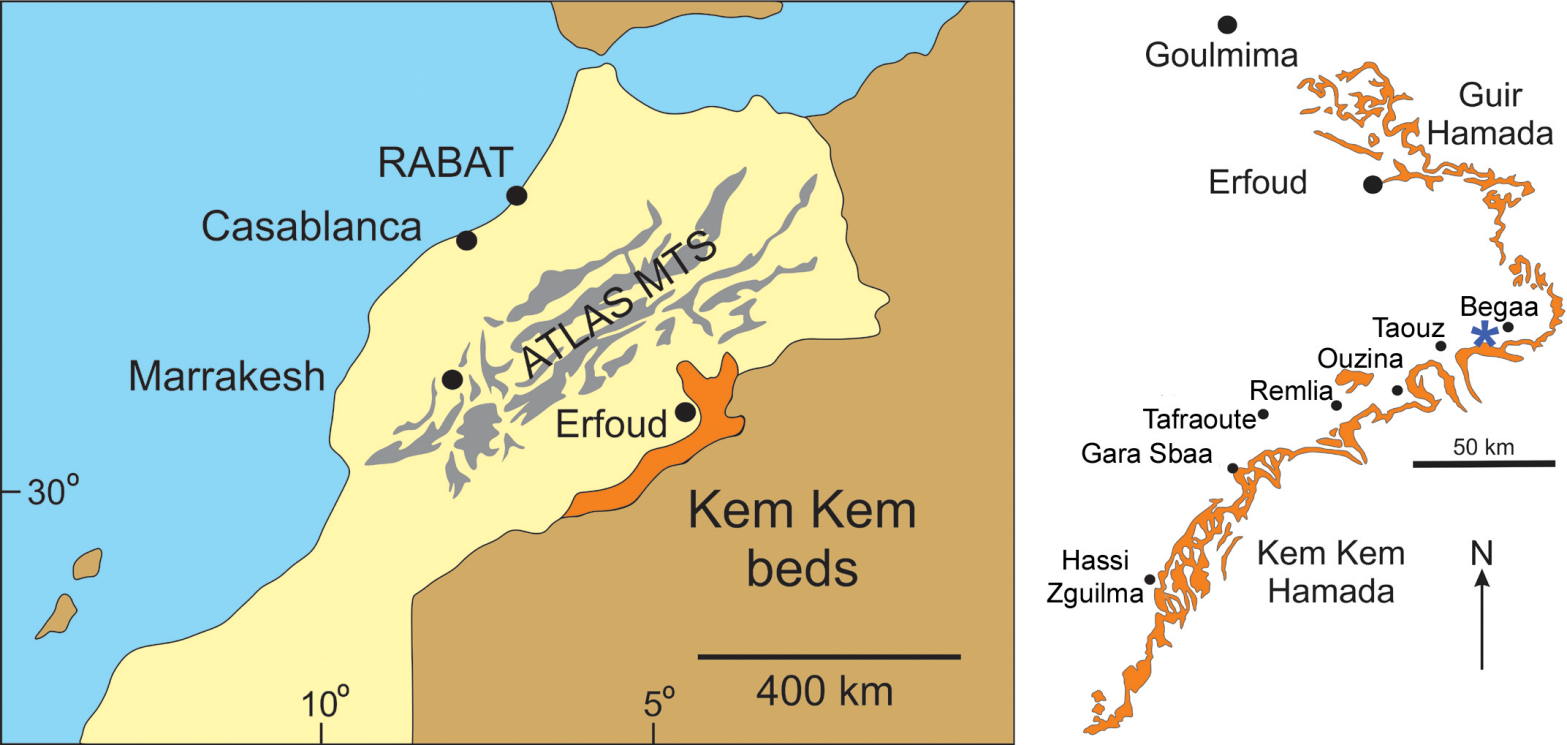
Geographic map of the fossil location, Morocco. Geographic map of the fossil location South of Erfoud, between the villages of Taouz and Begaa, Errachidia Province, Morocco; the main localities and landscape elements cited in the text are shown in the map. The asterisk marks the site from where the specimen MSNM V6894 was collected (modified from Ibrahim et al., 2016).

In recent decades, several theropod remains have been reported from the mid Cretaceous of North Africa (Sereno et al., 1994, 1996, 1998, Sereno, Wilson & Conrad, 2004; Russell, 1996; Riff et al., 2004; Dal Sasso et al., 2005; Mahler, 2005; Novas, Dalla Vecchia & Pais, 2005; Brusatte & Sereno, 2007; Sereno & Brusatte, 2008; D’Orazi Porchetti et al., 2011; Ibrahim et al., 2014; Chiarenza & Cau, 2016; Hendrickx, Mateus & Buffetaut, 2016). Most have been referred to abelisauroids and basal tetanurans (i.e., allosauroids and spinosaurids) of medium to large body size. In May 1999, the Museo di Storia Naturale di Milano, in collaboration with the Geological Service of Morocco and with the logistical support of G. Pasini (Appiano Gentile, Italy), carried out a palaeontological expedition in the southern part of the Errachidia Province, Morocco, focusing on invertebrate fauna (Alessandrello & Bracchi, 2003). Some prospecting was also carried out in the Tafilalt region, near Erfoud. Among fossil finds, there was an almost complete, very small pedal ungual, surface collected by Pasini to the South of Erfoud, between the villages of Taouz and Begaa (Fig. 1). This specimen remained unnoticed in the Vertebrate Paleontological Collection of the Museo di Storia Naturale di Milano, until the recent discovery of a new partial skeleton of *Spinosaurus aegyptiacus* published by Ibrahim et al. (2014), which preserves an almost complete right pes with peculiar pedal ungual morphology. The striking similarities with the pedal unguals of *Spinosaurus* allowed us to refer the isolated specimen to this genus, of which it represents the smallest individual reported up to today. This fact is even more remarkable, considering the dramatic size attained by some large specimens of *Spinosaurus*, which are possibly the longest, and among the largest theropod dinosaurs ever found (Dal Sasso et al., 2005; Ibrahim et al., 2014).

## Materials and Methods

The specimen described herein was legally collected and transported to Italy together with the material published by Alessandrello & Bracchi (2003), in agreement with the Geological Service of Morocco, and permanently deposited in the Vertebrate Palaeontological Collection of the Museo di Storia Naturale di Milano (MSNM V), where it is catalogued as MSNM V6894. In conformity with Weishampel, Dodson & Osmólska (2004), we adopt the following anatomical terms of the *Nomina Anatomica Veterinaria* (NAV 1994) and the *Nomina Anatomica Avium* (NAA 1993): plantar (opposite to the back), dorsal (toward the back), proximal (toward the mass of the body), and distal (away from the mass of the body). Specimen images were taken with a Canon PowerShot S50, mounted on an ocular tube attached to a Leica MS5 stereomicroscope with Plan Apo 1.0× objective and carrier AX, then stacked with software Combine ZP.

**Systematic Palaeontology**
DINOSAURIA Owen, 1842
THEROPODA Marsh, 1881
SPINOSAURIDAE Stromer, 1915
SPINOSAURUS Stromer, 1915
cf. *Spinosaurus aegyptiacus* Stromer, 1915

MSNM V6894 strongly resembles the pedal ungual phalanges associated to diagnosable skeletal remains of specimen FSAC-KK18888, described by Ibrahim et al. (2014) and defined as the neotype of *S. aegyptiacus*. MSNM V6894 shares with FSAC-KK18888 the following diagnostic characters: pedal unguals with flat plantar surface; pedal unguals broader than deep with length almost four times of the proximal depth. The overall morphology, proportions, and pattern of furrows are also very similar (see “Description and comparisons”). Following Ibrahim et al. (2014) we refer the ungual MSNM V6894 to cf. *S. aegyptiacus*. The variability found in cervicodorsal vertebrae (Evers et al., 2015) and quadrates (Hendrickx, Mateus & Buffetaut, 2016) might indicate a higher diversity among the spinosaurid material from the Albian–Cenomanian of North Africa than previously recognized. This proportional and morphological diversity may be related to individual variability or sexual dimorphism, or it could be above the species level. However, taking into account the low number of the known specimens, their low degree of completeness, their apparently strict taxonomic affinities, their occurrence in the same strata (or, more often, their uncertain stratigraphic provenance), and all the difficulties and controversies in investigating these aspects and, ultimately, in defining a species in palaeontology, we prefer to regard all the spinosaurid material (including pedal unguals) from the Kem Kem Beds as belonging to cf. *S. aegyptiacus*, pending more complete, articulated remains and reliable geological data. Further comments on this topic are beyond the purpose of this paper.

**Locality:** MSNM V6894 comes from some kilometers south of Erfoud, between the villages of Taouz and Begaa, Errachidia Province, Morocco (Fig. 1).

**Horizon:** Kem Kem Beds, Cenomanian, Upper Cretaceous (Sereno et al., 1996). The specimen was collected by G. Pasini (2008, personal communication) together with rostral teeth of the Aptian–Cenomanian elasmobranch *Onchopristis* sp. (Rage & Cappetta, 2002; Russell, 1996).

### Description and comparisons

**Figure 2:**
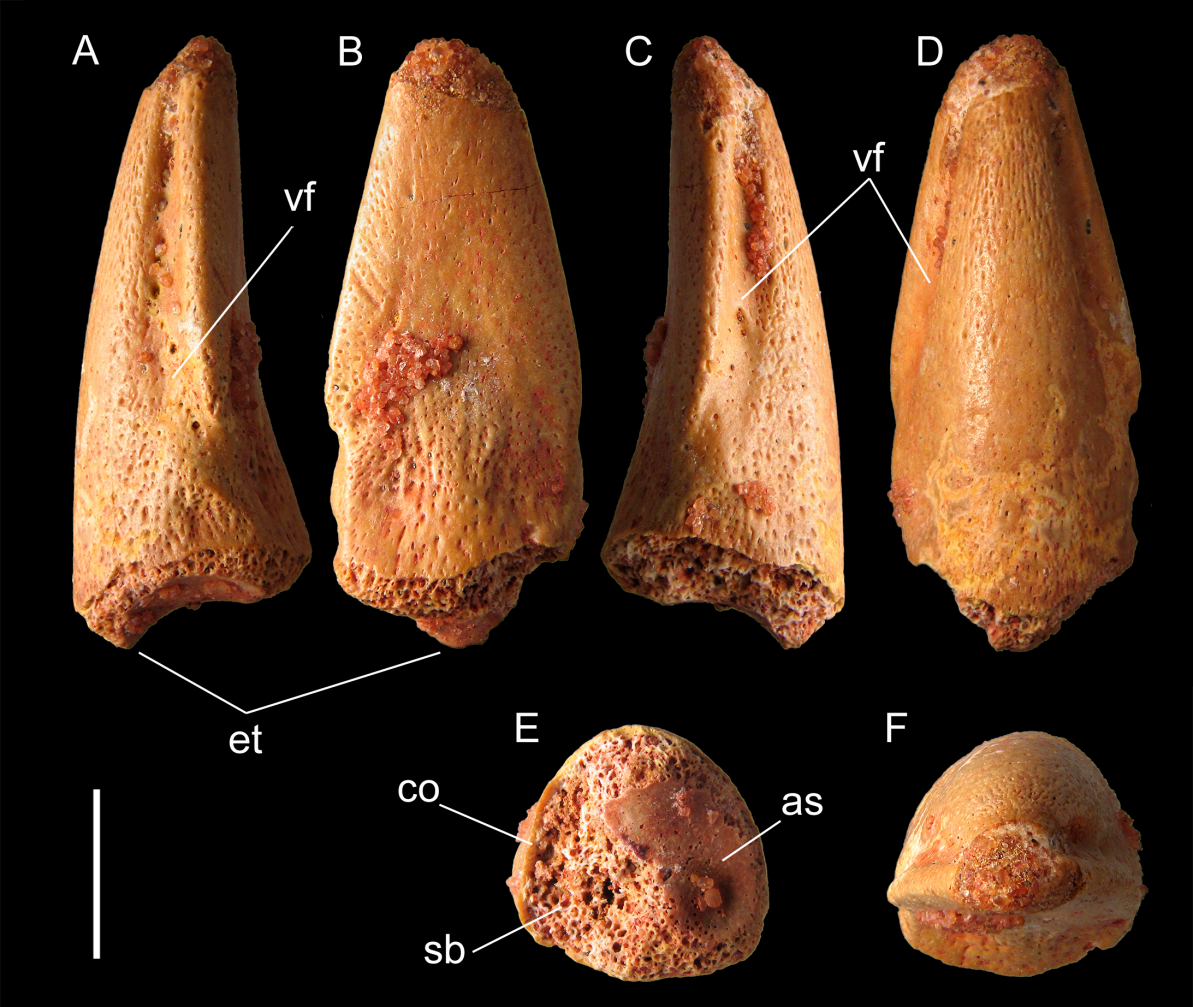
Pedal ungual phalanx of *Spinosaurus*. Specimen MSNM V6894 in right lateral (A), left lateral (B), dorsal (C), plantar (D), proximal (E), and distal (F) views. Abbreviations: as, articular surface; co, cortex; et, extensor tubercle; sb, spongy bone; vf, vascular furrow. Scale bar equals 5 mm. Photos by M. Zilioli, used with his permission.

**Figure 3:**
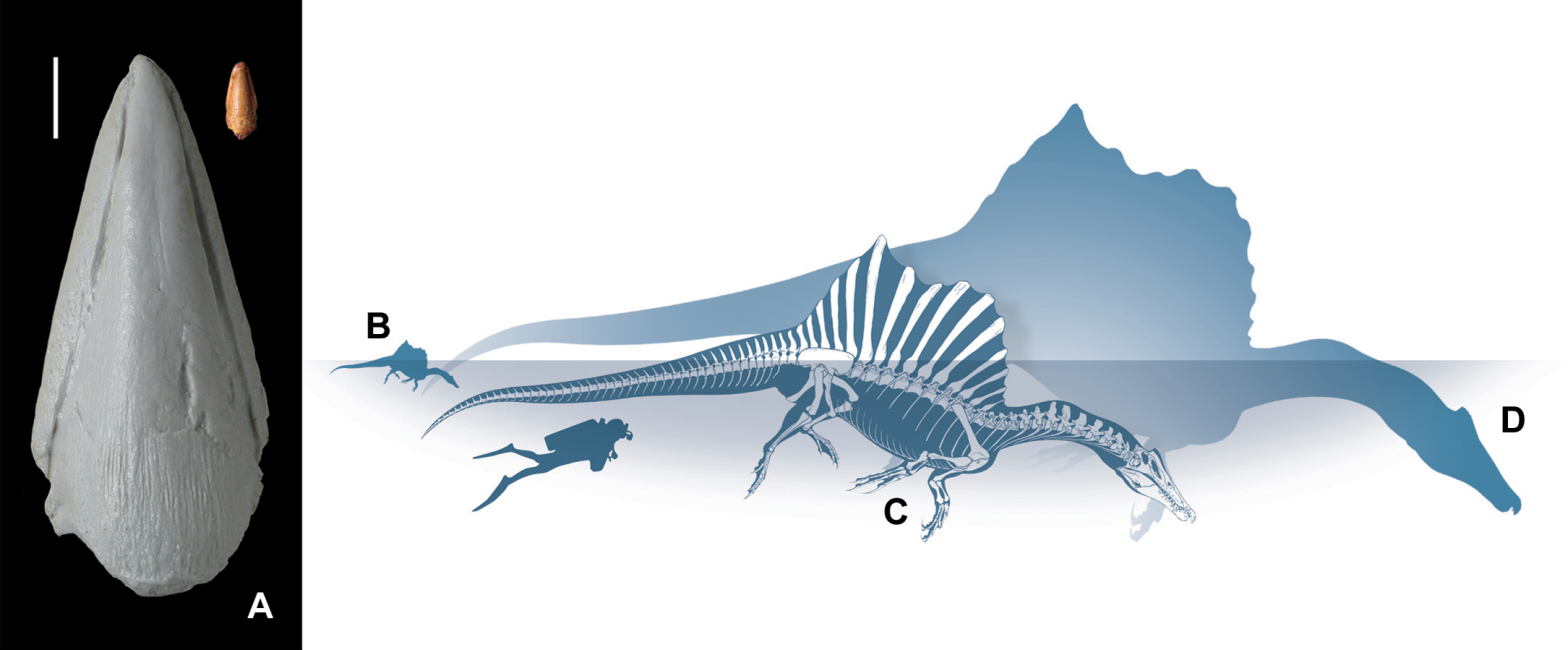
Anatomical and size comparisons. Specimen MSNM V6894 in dorsal view, compared to a cast of the right ungual III-4 of specimen FSAC-KK18888 (A). Size-comparison of selected *Spinosaurus* individuals from the Kem Kem Beds: MSNM V6894 (B, this paper), the neotype FSAC-KK18888 (C) and the largest known individual MSNM V4047 (D), compared with *Homo* (1.75 m tall). Drawing by Marco Auditore and Prehistoric Minds, used with their permission. Scale bar equals 20 mm in A. Photos by M. Zilioli and C. Dal Sasso, used with their permission.

The specimen MSNM V6894 is almost complete, except for the distalmost 2 mm of the tip and most of the proximal articular surface (Fig. 2). Sediment remains are inset in the bone pits and cemented in a few small patches on the bone surface, documenting the provenance of the specimen from a fine grained sandstone layer. The general morphology (i.e., shape, proportions, pattern of furrows) of MSNM V6894 strongly recalls that of the pedal ungual phalanges of the partial skeleton FSAC-KK18888, that are articulated to the rest of the pes (Fig. 3A; Ibrahim et al., 2014: fig. S1), and that of three isolated unguals, the specimen MPCM 13574 (Novas, Dalla Vecchia & Pais, 2005: fig. 2), the lost specimen Nr. 1922 X45 “*Spinosaurus* B” (Stromer, 1934: taf. I, figs. 17a–b; Ibrahim et al., 2014: fig. S2D), and the unlabelled ungual reported by De Lapparent (1960: pl. VI, figs. 10–12) and erroneously referred to *Carcharodontosaurus*. All these unguals are nearly flat ventrally and greatly differ from the deeper recurved unguals of many other Mesozoic theropods (Novas, Dalla Vecchia & Pais, 2005; Maganuco et al., 2007; Maganuco, Cau & Pasini, 2008). Among the pedal unguals of the specimen FSAC-KK18888, the ungual of digit III is the most similar to MSNM V6894, due to its proportions and symmetry. Based on these apparent features, we then refer MSNM V6894 to a pedal digit III.

The shape of this bone is slightly and continuously convex along its dorsal margin, whereas its plantar surface is almost flat, especially on the preserved distal half of the bone, thus resulting low and elongate in lateral aspect, and with sharply defined margins separating the plantar surface from the medial and lateral sides. Both the lateral and medial surfaces are slightly convex and bear a single, unforked vascular furrow extending for most of the ungual length; these collateral furrows match the faint ungual curvature in lateral aspect, and are located close to the plantar margin of the bone, flowing into the plantar surface proximally. Two shallow semicircular depressions exist on the proximal end of the plantar surface, as in MPCM 13574 (Novas, Dalla Vecchia & Pais, 2005: fig. 2C) and FSAC-KK18888. The depressions are separated by a low median bump wider than the ridge that can be seen in MPCM 13574 and in some pedal unguals of FSAC-KK18888. A distinct flexor tubercle is absent on the plantar surface. In proximal view, the bone is as tall as broad. The proximal articular surface is not well-preserved but it is faintly divided in two sub-equal articular surfaces, it being slightly convex along the midline and slightly concave on the preserved right side. The base of the proximodorsal lip (extensor tubercle) is robust but the rest of the lip is eroded. Its preserved portion projects proximally overhanging the proximal articular surface and it slopes proximodorsally forming an angle of about 45° respect to the plantar surface of the bone. Numerous foramina are present. The collateral furrows exhibit either the smoother surface or the largest foramina, which are more numerous in the right lateral furrow.

### Ontogenetic assessment

The ungual MSNM V6894 shows several features that indicate skeletal immaturity. The bone surface is densely pitted, not only towards the distal tip (Fig. 2C), but also all along its lateral and plantar sides/walls, whereas in the unguals of the subadult FSAC-KK18888, nutrition pits are retained only within the collateral furrows. A porous texture, with even more dense furrows and pits producing a “scarred” effect, is found in archosaur hatchlings (Sanz et al., 1997; Horner, de Ricqlès & Padian, 2000; de Ricqlès et al., 2000), and disappears gradually during growth, as it can be seen in immature extant crocodilians (C. Dal Sasso & S. Maganuco, 2010, personal observation), and birds (L. M. Chiappe, 2006, personal communication). The scarred effect is marked on the limb bones of the perinate and juvenile theropods, such as *Scipionyx* (Dal Sasso & Maganuco, 2011), *Sinornithoides* (Currie & Dong, 2001), *Juravenator* (Göhlich & Chiappe, 2006), the young adult *Sinosauropteryx* (Currie & Chen, 2001), and extant birds (Tumarkin-Deratzian, Vann & Dodson, 2006; Watanabe & Matsuoka, 2013).

In specimen MSNM V6894, the interior of the bone is also highly porous, as is well exposed in proximal view (Fig. 2E). Under the cortex, which is limited to a 600–800 μm thick layer, there are thin pillars of bone, with a honeycomb arrangement delimiting large trabecular spaces. This indicates that the spongy bone was highly spongy and vascularized. In sum, taking into account the degree of “scarring” and porosity of the bone, we estimate that the ungual MSNM V6894 pertained to a very young, but not perinate spinosaurid.

The measurements of MSNM V6894, compared to those of the ungual phalanges of FSAC-KK18888 (Table 1), in primis III-4 R, indicate fundamental growth isometry for these bone elements. Minor morphological difference exists. For instance, the proximal articular surface is slightly taller dorsopalmarly than wide mediolaterally, approaching the compression index of the ungual that in adults occupy different (internal) positions in the pes (e.g., I-2 and II-3 R in FSAC-KK18888); and the bone margins (or bony carinae) underlying the collateral furrows do not possess the arrow-like, backward pointed apices seen in some adult specimens (Stromer, 1934: taf. I, figs.17a–b).

**Table 1:** Basic measurements.

Selected measurements expressed in mm	MSNM V 6894	FSAC-KK18888				
		I-2 R	II-3 R	III-4 R	IV-5 R	?IV-5 L
Maximum length	21* (18.7)	94	118	130	87	93
Proximal dorsopalmar diameter	7.5	37	37	36	34	35
Proximal mediolateral diameter	7.1	32	40	48	37	40
Midlength dorsopalmar diameter	5.6	25	26	25	22	24
Midlength mediolateral diameter	7.0	30	35	46	30	34
Elongation index (maximum length/proximal mediolateral diameter)	2.95	2.93	2.95	2.70	2.35	2.32
Compression index (proximal dorsopalmar diameter/proximal mediolateral diameter)	0.94	1.15	0.92	0.75	0.91	0.87

**Notes:**

Basic measurements of specimen MSNM V6894 and of the pedal unguals of specimen FSAC-KK1888. Asterisks indicate estimated measurements; brackets indicate incomplete measurements.

Recently, Curry Rogers et al. (2016) found that the titanosaurian sauropod *Rapetosaurus*, in spite of massive changes in body size, maintained isometric relationships in the limb bones throughout ontogeny, which indicates an active precocial growth strategy. Similarly, Mateus, Antunes & Taquet (2001) documented that the proportions of presacral vertebral centra, between embryos and adults of the theropod *Lourinhanosaurus,* are identical and even superposing in published graphs. On the other hand, non-phalangeal elements of theropod hind limbs are reported to show significant allometry (Holtz, 1995; Christiansen, 1998; Currie, 2003), affecting limb proportion and cursorial potential.

## Conclusion

The specimen described here improves the knowledge about the appendicular skeleton of the spinosaurid theropods from the Kem Kem Beds (Late Cretaceous of Morocco) published by Ibrahim et al. (2014).

The new material indicates that the pedal ungual phalanges of *Spinosaurus* grew with isometry and it documents the smallest individual referable to *Spinosaurus*, a genus/taxon usually indicated as the longest if not the largest theropod dinosaur (Dal Sasso et al., 2005). The specimen FSAC-KK18888, with an estimated body length of 11 m, has an ungual phalanx of digit III that is 130 mm long. Assuming isometry—although isometrical scaling of the other parts of the spinosaur hind limb skeleton shown in Fig. 3 must be considered as tentative—the 21 mm long ungual MSNM V6894 would pertain to an early juvenile individual, 1.78 m long (Figs. 3B–3D), that is half the estimated length of the smallest *Spinosaurus* published up to date, represented by the isolated quadrate MNHN KK374 (Hendrickx, Mateus & Buffetaut, 2016).

According to Ibrahim et al. (2014), the unguals in *Spinosaurus* are reminiscent of the flattened pedal unguals of shorebirds that do not perch (Manegold, 2006), and the whole foot may have been adapted to traversing soft substrates or webbed for paddling. We agree with this hypothesis although it needs to be tested in the future based on more complete fossil remains and biomechanical analyses. The isolated tiny ungual here referred to a small, early juvenile of *Spinosaurus* indicates that the pes had the same locomotor adaptations observed in large individuals, that were probably achieved early in ontogeny and retained for the entire lifespan.

## Abbreviations

FSAC: Faculté des Sciences Aïn Chock, Casablanca, Morocco
MHNM: Muséum d’Histoire Naturelle of Marrakech, Morocco
MPCM: Museo Paleontologico Cittadino di Monfalcone, Gorizia, Italy
MSNM: Museo di Storia Naturale di Milano, Italy.

## Additional Information and Declarations

### Data Availability

The following information was supplied regarding data availability: The fossil specimen MSNM V6894 described in this manuscript is permanently deposited in the appropriate repository (i.e., the Vertebrate Palaeontological Collection of the Museo di Storia Naturale di Milano, MSNM V) and is accessible to other researchers. The MSNM is a public permanent inalienable institution, and is part of the “POLO DEI MUSEI SCIENTIFICI” of the Comune di Milano, together with the Planetarium and the Aquarium (see the MSNM webpage: https://www.comune.milano.it/dseserver/webcity/Documenti.nsf/webHomePage?OpenForm&settore=MCOI-6C5J9V_HP). The POLO is among the institutions officially certified by the government of Regione Lombardia to possess all requirements in order to be named a “museum.” In addition, the MSNM is also a member of ICOM, therefore it follows all ICOM guidelines.
